# The Effectiveness of Linear and Nonlinear Pedagogical Approaches in Team-Invasion Ball Sports: A Systematic Review

**DOI:** 10.1186/s40798-025-00893-y

**Published:** 2025-08-04

**Authors:** Liam Bromilow, Nikki Milne, Carl T. Woods, Caroline K. Dowsett, Justin W. L. Keogh

**Affiliations:** 1 Faculty of Health Sciences and Medicine, Bond University, Gold Coast, Australia; 2 School of Human Movement and Nutrition Sciences, The University of Queensland, St Lucia, Australia; 3 Sport and Exercise Science, College of Healthcare Sciences, James Cook University, Townsville, Australia; 4 Sports Performance Research Centre New Zealand, Auckland University of Technology, Auckland, New Zealand; 5 Kasturba Medical College, Mangalore, Manipal Academy of Higher Education, Manipal, Karnataka India

**Keywords:** Skill acquisition, Coaching science, Metatheory, Constraints manipulation

## Abstract

**Background:**

In the sport sciences, skill development is often (implicitly) explained through two metatheoretical perspectives: *interactionism* and *transactionism*. Given certain assumptions, the former adheres to a linear pedagogical approach to learning, while the latter follows a nonlinear pedagogical approach. The aim of this systematic review was to compare the effects of linear and nonlinear pedagogical approaches on the development of technical and tactical skills in team-invasion ball sports.

**Methods:**

A systematic search of six databases (EmBase, PubMed, SPORTDiscus, OVID Medline, CINAHL, and OVID PsychInfo) was undertaken from root to 1st May 2024. Included studies were critically appraised using the ROBINS-I and RoB2 instruments. A narrative and descriptive synthesis approach was utilised.

**Results:**

From 7450 potential records, nine studies were included, which explored the effects of a nonlinear versus linear pedagogy for developing technical and tactical skills in team-invasion ball sports. While, for most outcomes, the results showed there were no significant differences, nonlinear pedagogy did appear more favourable in 34% of technical outcomes. Further, descriptive synthesis of four studies revealed that nonlinear pedagogy was significantly better for developing tactical skills in 66% of outcomes.

**Conclusions:**

While based on limited studies, linear and nonlinear pedagogical approaches appear to achieve similar results with regards to technical skill development. However, nonlinear pedagogy was favoured in some studies. With regards to tactical skill development, nonlinear pedagogical approaches appear better than linear approaches. Further high-quality research is needed to confirm these findings and examine how they may be implicated by the representativeness of the assessment instruments.

**Key Points:**

- Both linear and nonlinear pedagogical approaches assist with skill development in team-invasion ball sports.
- Nonlinear pedagogical approaches generally result in greater effects when developing tactical skills, while most literature shows there are no significant differences between approaches when developing technical skills.
- Further high-quality research exploring the effects of these pedagogical approaches is required to substantiate these findings.
- Questions remain regarding the representativeness of the assessment instruments used in the studies included in this review.

**Registration:**

This systematic review is registered with Open Science Framework- https://osf.io/za247/

**Supplementary Information:**

The online version contains supplementary material available at 10.1186/s40798-025-00893-y.

## Background

The development of skill is of paramount concern to coaches, practitioners and athletes across the sporting landscape. Accordingly, understanding the process through which skills are developed is essential to supporting a team and player’s potential for success in sport [1]. With that in mind, it is critical for sports coaches to understand the required pedagogical practices to support athlete skill development.

To help elucidate such pedagogical practices, we feel it is important to start at a *metatheoretical* level. Briefly, by metatheoretical, we mean a group of theoretical approaches that share common assumptions and commitments [2, 3], and which inherently shape the way in which people inquire about the world [2]. The first of relevance, here, is the *interactionist* metatheory [2], which fosters a mechanistic and linear view of causality, most noted through input–output models of behaviour [3]. A key commitment of this metatheory is a unit of analysis scaled to the individual, viewed as an entity that exists separate to a surrounding. A manifestation of this unit of analysis are information-processing models that imbue a linear sequence of causation–that is, an input stimulus from the environment is processed in the mind, leading to the output of a response by the body [2, 4, 5]. Thought of as a ‘closed-loop’ process, the brain receives a stimulus (information) through the body’s sensory organs, and then sends a message through the central nervous system to enact a stored motor program [5]. Depending on an individual’s stage of development [6], the resultant movement requires a certain level of cognition to elicit a response. In this sense, interactionism posits that the control, coordination and *acquisition* of skill is ultimately centred on an individual’s ability to process information extracted from one’s surround [7].

Some argue, however, that there is a misnomer here. Namely, skills may not be *acquired* per se, but are rather continually *adapted* through the constant interaction of a variety of constraints [8]. This leads to the second metatheory of relevance–that of *transactionism* [2]. Differing to interactionism, a key commitment of this metatheory is a scale of analysis distributed across the performer-environment system. In other words, movement and its subsequent control is seen to arise from the dynamic relation sustained between a performer and environment [9]. A contemporary, extent perspective of this metatheory is described within an ecological dynamics framework [10], which brings together key ideas from dynamical systems theory and ecological psychology [10] to explain the control and coordination of movement as a deeply embodied and embedded phenomenon distributed across the performer-environment relation [11, 12].

The implications of these respective metatheoretical approaches–and their extant perspectives–are profound for the sport sciences generally, and the field of skill development specifically. For example, in following an information processing approach, grounded in the metatheory of interactionism, a coach would likely prioritise high-levels of continued repetition and instruction, verbal cues, and feedback anchored to the acquisition of a ‘correct’ motor program [13]. Learning, in this view, is about the consolidation of the purported ‘correct’ motor program such that it is stored in, and retrieved from, the long-term memory when required. Given the sequential nature of this approach, it has been described as being a ‘linear pedagogy’, outlined by four key principles: *idealistic movement patterns, simplified movement sequences, limited movement variability,* and *internal focus of attention* [14]. In contrast, by following an ecological dynamics rationale, grounded in the metatheory of transactionism, a coach’s role is reconceptualised as a designer of practice tasks that prioritises the performer-environment relation [15]. An important feature of this relationality is its nonlinearity [16], which means learning is not a sequential process. As such, proponents of an ecological dynamics rationale follow a ‘nonlinear pedagogy’, in which performers are encouraged to search, discover and explore solutions to various movement problems [16, 17]. Broadly, the components of nonlinear pedagogy include: *task representativeness*, *task simplification*, *constraints manipulation*, *functional variability*, and *external focus of attention* [18].

Not only is appreciating metatheoretical roots important here, but considering what is meant by the term ‘skill’ is worth a brief note. In generalist terms, skill refers to any goal-directed activity that one learns through practice and experience [19]. Within the sport sciences, this generalist perspective has typically been demarcated further, with skills being classified as either ‘technical’ or ‘tactical’. Broadly, technical skills include those requiring some executed outcome–such as a place kick in rugby or a free throw in basketball, while tactical skills oft-refer to game sense and decision-making [20–22]. While this demarcation may reflect sport science’s implicit reliance upon the interactionist metatheoretical tradition (i.e., seeing ‘skill’ as a reduced construct localised to the inner workings of the performer [2]), our concern, here, is less about critiquing this classification of skill, and more about investigating how each have been developed through the use of linear or nonlinear pedagogical approaches. To this end, a pertinent question arises: which pedagogical approach–linear or nonlinear–is more effective for supporting the development of skill in sport?

Indeed, a number of published reviews have been conducted in response to such a question–see, for example, Bergmann et al. [23] and Clark et al. [24]. However, these reviews reflect either a single sport or have focused on a single component of nonlinear pedagogy. To date, no systematic review has been conducted that directly compares linear and nonlinear pedagogical approaches in a team-invasion ball sport context. The aim of this systematic review was to critically evaluate literature exploring the relative effectiveness of linear and nonlinear pedagogies for supporting skill development in team-invasion ball sports.

## Methods

This systematic review followed the Preferred Reporting Items for Systematic Reviews and Meta-Analyses (PRISMA) guidelines [25]. The review was registered prospectively with the Open Science Framework on 1st November 2023 (https://osf.io/za247/) prior to data extraction.

### Eligibility Criteria

The inclusion and exclusion criteria used in this study can be found in Table 1.

**Table 1:** Eligibility criteria of systematic search

Inclusion criteria	Exclusion criteria
Studies relating to team invasion ball-sports, published in full text, in English language, in peer reviewed academic journals Quantitative study approaches using intervention/experimental designs with an appropriate control/comparison group (e.g., Randomised Controlled Trials (RCTs), Pseudorandomised controlled trials, comparative studies with concurrent controls (i.e., non-randomised trials, cohort studies (prospective or retrospective) Must clearly investigate linear and nonlinear pedagogical methods Intervention duration of at least two weeks, or at least four sessions, to account for chronic adaptations Years considered–Database inception to May 2024 (i.e. no restriction on year of publication)	Studies that implemented other non-traditional pedagogical approaches such as Teaching Games for Understanding (TGfU), Differentiated Learning (DL), and Tactical Games Approach (TGA) Studies in which pedagogical approaches cannot be clearly discerned through the methodology Studies that do not provide statistical outcomes (means, standard deviation, confidence intervals) Studies that do not use original data

### Information Sources

A systematic search was conducted from root to 27th September 2023, using six databases: EmBase, PubMed, SPORTDiscus, OVID Medline, CINAHL, and OVID PsycINFO. The search strategy was developed in consultation with an experienced health sciences and medicine faculty librarian for all databases. A forward and backward citation analysis of included studies was also performed to identify any additional studies not captured through the original search. An updated systematic search was conducted on 1st May 2024 for articles published between 27th September 2023 and 1st May 2024.

### Search Strategy

Utilising the PICO (Population, Intervention, Comparison/Control, Outcome) format, the search strategy combined keywords/text words and MeSH terms related to linear and nonlinear pedagogy in a coaching environment of team-invasion ball sports. The Polyglot Search Translator [26] automation tool was implemented to assist with the MeSH term coding across databases. The search terms for PubMed are listed below, with the search strategies for all databases being presented within the Supplementary Information Files:

(Rugby*[tiab] OR Soccer[tiab] OR Football[tiab] OR oztag[tiab] OR futsal[tiab] OR lacrosse[tiab] OR hockey[tiab] OR hurling[tiab] OR "Water Polo"[tiab] OR Basketball[tiab] OR Netball[tiab] OR Handball[tiab] OR Rugby[Mesh] OR Soccer[Mesh] OR Football[Mesh] OR Hockey[Mesh] OR Basketball[Mesh]) AND (coach*[tiab] OR teach*[tiab] OR mentor[tiab] OR train*[tiab] OR learn*[tiab] OR player[tiab] OR Teaching[Mesh] OR Mentors[Mesh] OR Learning[Mesh] OR "Physical Education and Training"[Mesh] OR athletes[Mesh]) AND ("non-linear pedagog*"[tiab] OR "non linear pedagog*"[tiab] OR "nonlinear pedagog*"[tiab] OR "constraints-led approach"[tiab] OR "constraints led approach"[tiab] OR "indirect instruct*"[tiab] OR "nonlinear dynamic"[tiab] OR "non-linear dynamic"[tiab] OR "linear pedagog*"[tiab] OR "direct instruct*"[tiab] OR linear[tiab] OR nonlinear[tiab] OR traditional[tiab]) AND (skill*[tiab] OR competence[tiab] OR capability[tiab] OR ability[tiab] OR perform*[tiab] OR develop*[tiab] OR precision[tiab] OR proficiency[tiab] OR technical[tiab] OR tactical[tiab] OR mechanism[tiab] OR motivation[tiab] OR prepar*[tiab] OR readiness[tiab] OR "Motor Skills"[Mesh] OR "Athletic Performance"[Mesh] OR Motivation[Mesh]).

### Selection Process

Titles and abstracts of each article were screened independently by two reviewers (LB and CD) for relevance according to the eligibility criteria. Records that fulfilled eligibility criteria at title and abstract level were then screened at full text level by the same reviewers. Reasons for exclusions at full text screening were documented for each report. Any discrepancies between reviewers were discussed and resolved, and a third reviewer (JK) was consulted when mutual consensus could not be achieved between the original reviewers. The outcomes of the screening process were documented using the PRISMA flow chart [25].

### Data Collection Process

The data extraction process was conducted by the first author and entered into a Microsoft Excel (Microsoft Corporation, v2404) spreadsheet for synthesis. All accessible information regarding study design, participant characteristics, intervention type, modality, statistical approach to analysis and main findings, including statistical significance, were extracted. Confidence in the body of evidence extracted from the included studies was ascertained by only including research papers that met Level II to III-2 of the National Health and Medical Research Council (NHMRC) Levels of Evidence Hierarchy [27]. Outcomes related to the progress of technical and/or tactical skills were of primary concern to this paper, being a pre-intervention and/or post-intervention score from an assessment instrument. Outcomes related to physical and physiological measures were not extracted as they were not relevant to the review questions. All data extracted were validated by a second reviewer (JK or CW) to ensure data accuracy. No automated data extraction tools were utilised. Where relevant data from individual studies were not published, the corresponding author was contacted with a request to provide the missing data (e.g., p-values).

### Critical Appraisal of Methodological Quality (including Risk of Bias) in Individual Studies

The methodological quality of individual studies was critically appraised by two reviewers (LB and CD) using the Cochrane Risk Of Bias In Non-Randomized Studies—Interventions (ROBINS-I) [28] and the Risk of Bias 2 (RoB 2) [29] for randomised trials. The ROBINS-I tool was implemented for studies that were non-randomised and utilised a robust criteria across seven domains, while the RoB 2 utilised five criteria to appraise randomised studies [28, 29]. These tools were chosen to critically appraise the individual studies due to the clarity of signalling questions to elicit information about the bias of the study design, conduct and reporting [28, 29].

To appraise each study, the reviewers used the signalling questions across the seven domains (ROBINS-I) or five domains (RoB 2) by answering ‘Yes’, ‘Probably Yes’, ‘Probably No’, ‘No’, or ‘No Information’. These responses were then added to the ROBINS-I or RoB 2 algorithm to provide a domain rating of *Low Risk*, *Some Concerns*, or *Serious Risk* (ROBINS-I)/*High Risk* (RoB2) for the risk of bias judgement [28, 29]. The study was deemed *Low Risk of Bias* if all domains were scored as Low; *Some Concerns of Bias* if at least one domain scored Some Concerns but there were no high concerns; and *Serious/High Risk of Bias* if at least one domain scored as High or multiple domains scored Some Concerns in a way that would substantially lower confidence in the result [28, 29]. If the two reviewers could not reach an agreement when validating the risk of bias score, a third reviewer was consulted (JK). The risk of bias summary tables were uploaded to the Risk of Bias Visualisation (robvis) data visualisation tool for representation [30].

### Data Synthesis (Including Measures of Effect)

The characteristics of all included studies were narratively synthesised to enable a comprehensive overview of the current literature. All studies were included in the data synthesis irrespective of risk of bias results. The outcomes of each study were categorised into two fields of development: 1) technical skill; or 2) tactical skill. Within each of these categories, data were reported as having a statistically significant effect or no effect from pre- to post-intervention measurements. This descriptive synthesis allowed a visual inspection of the collective evidence. Pre- to post-intervention mean change scores were utilised in this data synthesis process. All information relevant and available to the pre- and post-intervention data were recorded, including significant within-group and significant between-group differences. Pre–post change scores were also calculated for the within group effects so to more easily allow the reader to understand the changes within each group over time.

Significant differences in the effects of nonlinear and linear pedagogy were typically reported in studies using a variety of statistical tests such as repeated measures ANOVA that provided interaction Group x Time effects. A significant Group x Time interaction effect is interpreted as there being a significant difference between how one group changes over time compared to the other group–i.e. one group experiences a significantly bigger change than the other group. Where Group x Time interaction effects were not reported, statistical analyses comparing both groups at the post-intervention timepoint were used. Justification for this recommendation is described in the Cochrane Handbook for Systematic Reviews of Interventions [31]. Whilst the implementation of a meta-analysis was initially planned, it was not possible due to the heterogeneity of the study contexts, participants, and assessment instruments used to measure study outcomes. Therefore, a summary effect for pedagogical approaches to technical and tactical skill development was determined using a descriptive synthesis approach utilised elsewhere [32], whereby a summary outcome is documented as being effective ( +) if the collective results demonstrated ≥ 66% of outcomes favouring nonlinear pedagogical approaches. If 66% or more of the outcomes demonstrated no significant difference between linear and nonlinear approaches, the summary effect of (0) was assigned. If the threshold (≥ 66%) was not reached, a summary effect of inconclusive (?) was assigned. A sensitivity analysis of each descriptive synthesis was undertaken to explore the impact of including studies with serious risk of bias.

As is standard practice in sport science research, studies were expected to report on a range of outcome measures. When studies only reported inferential and sometimes descriptive statistical information for outcomes that did not designate significance between the nonlinear and linear groups, the first author of this paper attempted to contact the corresponding author for further information. If no response was received, outcomes where results were not documented were labelled as N.S. (Not Significant).

## Results

**Fig. 1:**
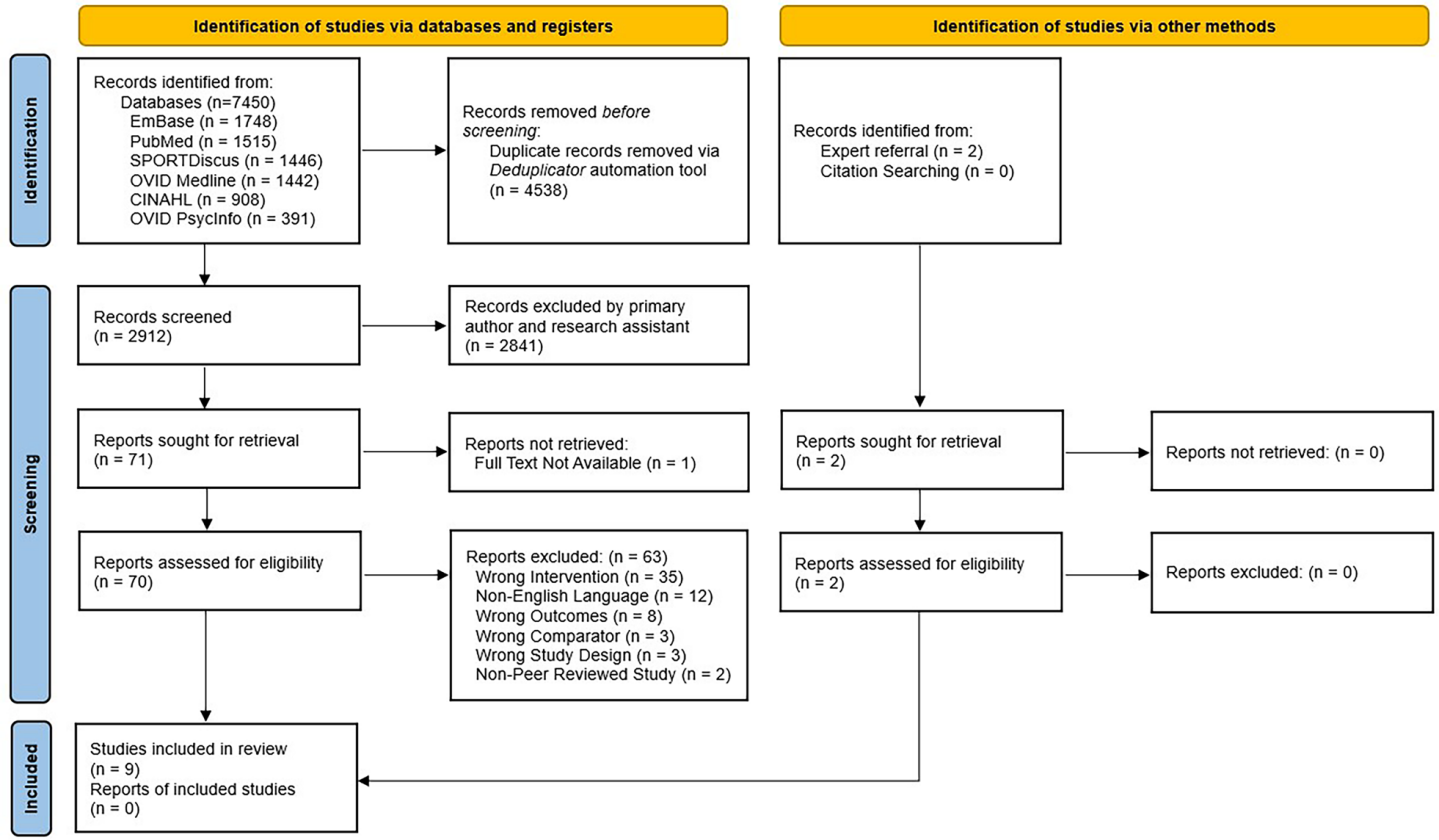
PRISMA flow chart

The initial search in 2023 yielded 6882 records, with a further 568 results in the supplementary search conducted in May 2024. This resulted in a total of 7450 identified studies, which were exported to EndNote X21 [33]. A Deduplicator automation tool (30) was then used to remove duplicates, with the final results manually screened by LB and CD to remove any further duplicates that were missed. After completion of the duplicate removal, 2912 records remained and were imported to the online platform Covidence (Covidence systematic review software, Veritas Health Innovation, Melbourne, Australia) for eligibility screening. Two additional reports were included for full-text screening based on expert referral, one of which did not appear in the original or supplementary search records, while the other was published after the searches were conducted. Further manual citation searching of the records did not provide any additional records. The PRISMA flow chart details the results of the selection process (Fig. 1).

Upon completion of the screening process, nine studies were included for data extraction. Almost half (n = 35) of the full text reports screened appeared to meet the inclusion criteria but were excluded due to theoretical differences in the lineage of their pedagogical approach. Other primary reasons for exclusions included being published in a non-English language, non-relevant outcomes (such as physiological measures), non-relevant comparator, incorrect study design, and not being published in a peer reviewed journal (see Supplementary Information Files). Key characteristics of the included studies are listed in Table 2.

**Table 2:** Key Characteristics of Included Studies

Study	Participants Age (yr.) Sex	Sport & Level	Skills Developed	Intervention Modality	Intervention Duration- Sessions (Weeks)	Skill Outcomes Assessed
Abate Daga et al. [35]	40 < = 9 M	Soccer Community-level soccer school	Ball Dribbling	NLP- Constraints Manipulation, Representativeness LP- Closed Drills	24 (12)	Technical
Bonney et al. [37]	22 22.3 ± 2.5 M	Australian Rules Football Amateur athletes player in a local senior competition	Kicking Handballing Movement with and without Pressure Catching	NLP- Constraints Manipulation, Functional Variability, Representativeness LP- Closed Drills	8 (4)	Technical Tactical
Cheong et al. [34]	70 19 to 27 48 M / 22F	Hockey Novice university students with no prior experience	Trapping Passing Shooting Dribbling	NLP- Constraints Manipulation, Functional Variability LP- Blocked practice, Repetition	6 (3)	Technical
Chow et al. [38]	224 12 to 13 M & F	Soccer Secondary school students. No mention of individual skill level	Passing Receiving Dribbling Kicking (Shooting) Defending	NLP- Constraints Manipulation, Representativeness, Functional Variability, Attentional Focus, Task Simplification LP- Repetition	16 (8)	Technical Tactical
Deuker et al. [39]	40 10 ± 1 M	Soccer Regional elite to regional sub-elite level competition	Passing Dribbling	NLP- Constraints Manipulation, Representativeness, Attentional Focus, Functional Variability LP- Closed Drills	10 (5)	Technical
Esposito et al. [42]	30 12 ± 1.2 No Information	Soccer Youth athletes at regional level	Passing	NLP- Constraints Manipulation, Attentional Focus, Functional Variability LP- Closed Drills, Repetition	24 (8)	Technical
Mohammadi Orangi et al. [40]	66 27.5 ± 2.7 M	Soccer Novice university students with no prior experience	Shooting Passing Trapping Dribbling Teamwork	NLP- Constraints Manipulation, Task Simplification LP- Blocked Practice, Repetition	24 (12)	Technical
Praxedes et al. [36]	19 11 to 12 M	Soccer Junior players participating in local sports league	Dribbling Shooting Passing Tactical Movements	NLP- Constraints Manipulation, Representativeness LP- Closed Drills, Blocked Practice	14 (7)	Technical Tactical
Roberts et al. [41]	22 16.4 ± 0.3 M	Soccer Youth academy from professional league club	Dribbling Shooting Passing Tactical Movements	NLP-Constraints Manipulation, Representativeness, Attentional Focus, Functional Variability LP- Closed Drills, Repetition	16 (4)	Technical Tactical

**Fig. 2:**
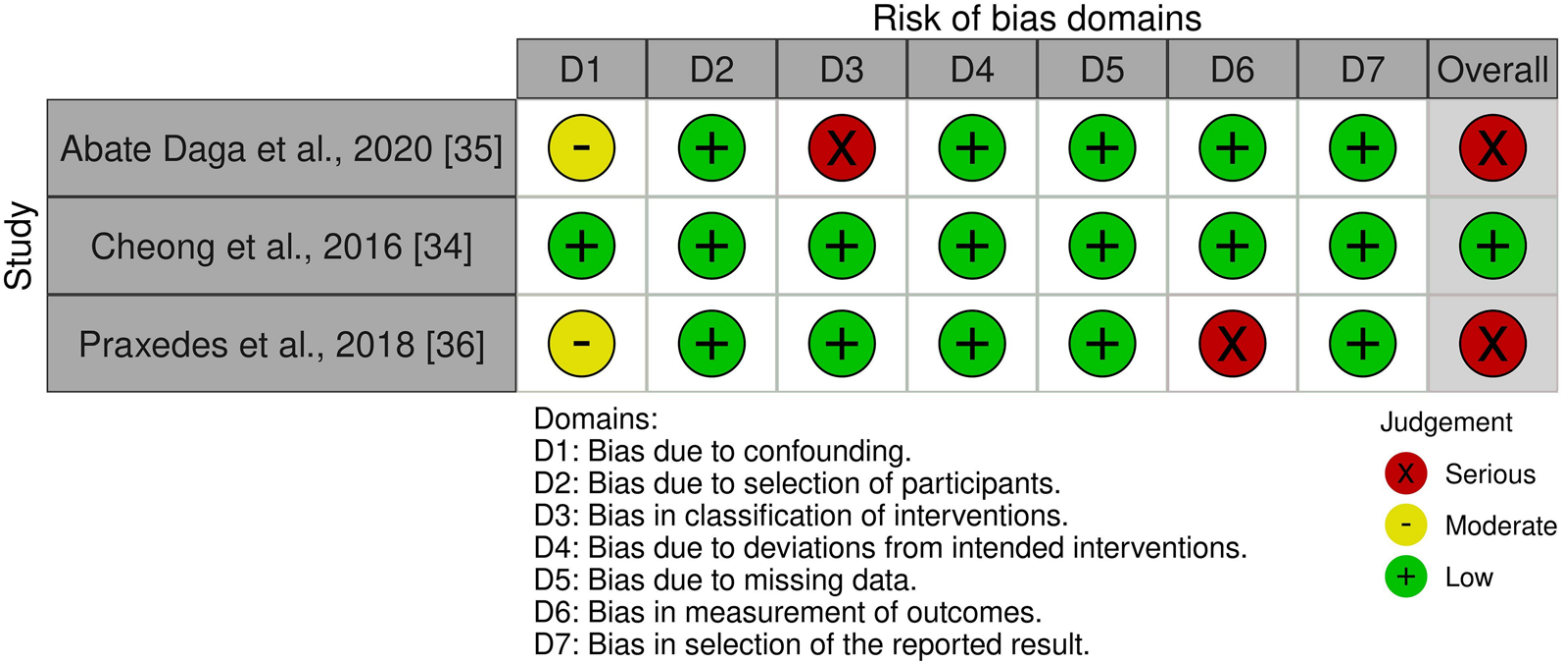
ROBINS-I traffic light summary

Using the ROBINS-I tool [28] for analysis of the risk of bias for non-randomised studies, Cheong et al. [34] presented as *Low Risk of bias* across all domains, while Abate Daga et al. [35] and Praxedes et al. [36] had an overall score of *Serious Risk of bias* (see Fig. 2). The latter two studies were rated as having *Serious Risk of bias* for selection of participants [35] and measurement of outcomes [36]. Regarding the selection of participants, the Abate Daga et al. [35] study was relegated to *Serious Risk* due to no information being provided about the domain question: *“Was classification of intervention status influenced by knowledge of the outcome or risk of the outcome?”* The Praxedes et al. [36] study was classified as *Serious Risk of bias* due to the measurements being taken from junior league soccer matches throughout a season, meaning there was no control over the consistency of opposition between the linear and nonlinear groups.

**Fig. 3:**
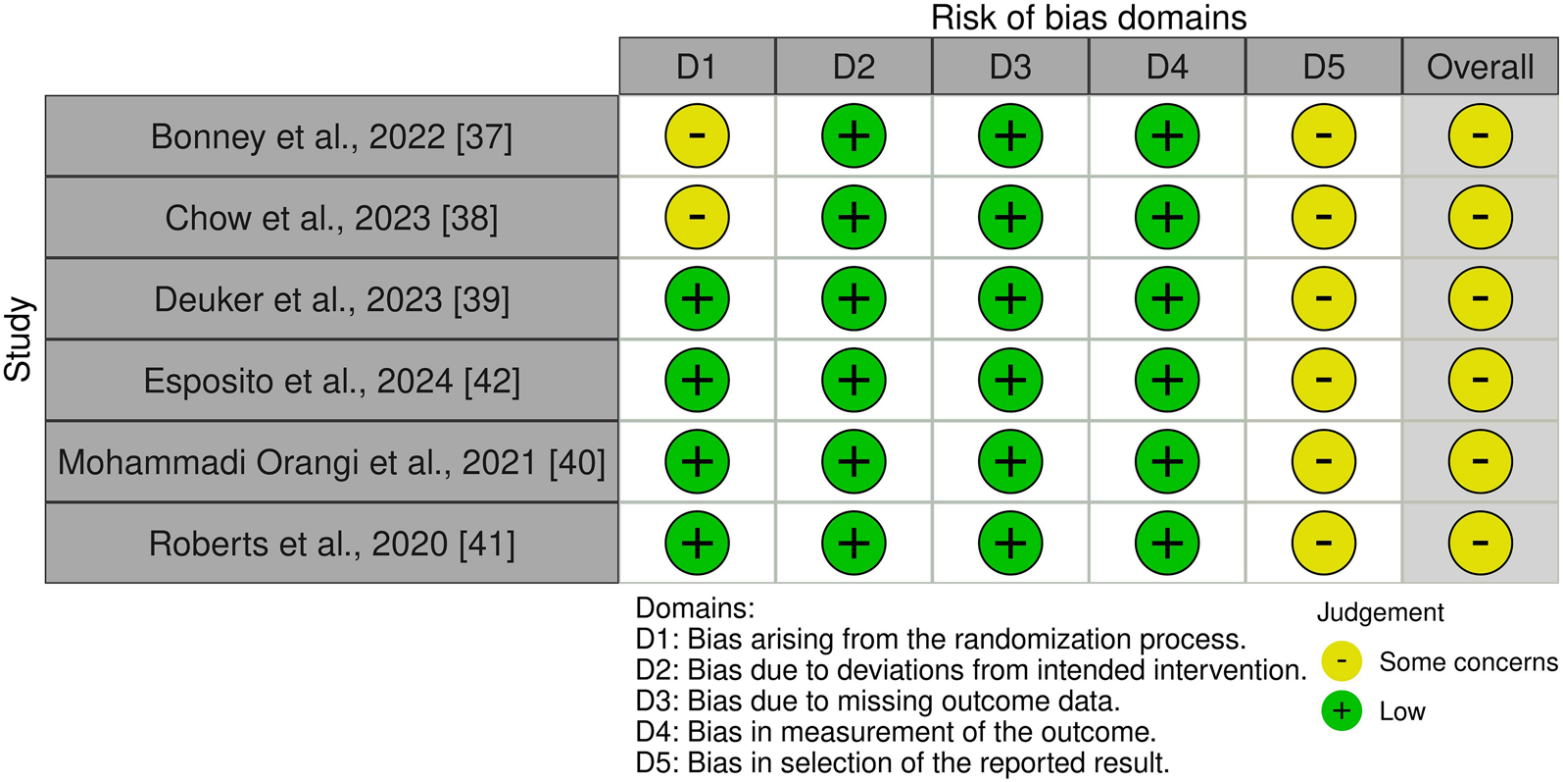
RoB 2 traffic light summary

The remaining six randomised studies were appraised for risk of bias using five domains from the RoB2 tool [29], with the outcomes represented visually in Fig. 3. All studies [37–42] were determined to have *Some Concerns* related to bias, due to bias in selection of reported results. Upon further analysis, this domain sought information regarding a ‘pre-specified analysis plan’, which the studies did not provide any information. The research team agreed that the studies did provide relevant information regarding the intentions of the data analysis for the present review. Further, Bonney et al. [37] and Chow et al. [38] were appraised as having *Some Concerns* for domain one: bias arising from the randomisation process, due to there being no information provided regarding the coordination or randomisation of the participants.

A summary of the key results for each study, including intervention duration (15.8 ± 6.6 sessions across 7.00 ± 3.2 weeks), pre- and post-test descriptive statistics and the relevant inferential statistics for all included studies are provided, showing that many technical and tactical outcomes in both the linear and nonlinear groups significantly improved from pre- to post-intervention testing (Table 3).

**Table 3:** The effects of nonlinear (NLP) and linear (LP) pedagogies to improve technical and tactical skills

Study	Variable	NLP Pre to Post Intervention (Mean ± SD) Δ = Mean Change Score *p-*value = within group difference	LP Pre to Post Intervention (Mean ± SD) Δ = Mean Change Score *p-*value = within group difference	Reported Between-Group Difference (*p-*value)
Abate Daga, et al. [35] (Soccer)	Shuttle Dribble Test (sec.)	Pre: 15.76 ± 1.129 Post: 12.75 ± 1.516 Δ -3.01,* p* = < 0.001	Pre: 14.00 ± 1.292 Post: 12.07 ± 0.965 Δ -1.93, *p* = < 0.001	*p* = 0.158
Bonney, et al. [37] (Australian Rules Football)	Kick Proficiency (Scale 1–5)	Pre: 56.20 ± 7.26 Post: 73.47 ± 10.00 Δ 17.27, *p* = < 0.010	Pre: 56.87 ± 16.61 Post: 59.50 ± 11.75 Δ 2.63, *p* = 0.950	*p* = 0.050
	Kick Distance (Count of times kicks executed for each distance)	0-20 m Pre: 6.36 ± 2.11 Post: 2.46 ± 0.93 Δ -3.90,* p* = < 0.001 20-40 m Pre: 2.73 ± 1.42 Post: 6.27 ± 1.79 Δ 3.54,* p* = 0.050 > 40 m Pre: 0.09 ± 0.30 Post: 0.36 ± 0.51 Δ 0.27, *p* = 0.530	0-20 m Pre: 3.00 ± 1.95 Post: 3.36 ± 2.01 Δ 0.36, *p* = 0.970 20-40 m Pre: 5.36 ± 3.85 Post: 5.18 ± 4.14 Δ -0.18,* p* = 1.000 > 40 m Pre: 0.36 ± 0.67 Post: 0.09 ± 0.30 Δ -0.27,* p* = 0.530	*p* = < 0.001 *p* = 0.050 *p* = 0.060^#^
	Time Before Skill Execution (Count of times skill execution occurred for each time block)	< 1 s Pre: 0.64 ± 0.81 Post: 2.27 ± 2.10 Δ 1.63, *p* = 0.040 1-2 s Pre: 3.00 ± 1.61 Post: 4.55 ± 1.51 Δ 1.55, *p* = 0.450 2-4 s Pre: 3.00 ± 1.00 Post: 3.46 ± 1.75 Δ 0.46, *p* = 0.960 > 4 s Pre: 2.46 ± 1.51 Post: 0.82 ± 0.98 Δ -1.64, *p* = 0.020	< 1 s Pre: 1.64 ± 1.29 Post: 0.82 ± 1.08 Δ -0.82,* p* = 0.530 1-2 s Pre: 4.27 ± 3.17 Post: 3.09 ± 2.91 Δ -1.18, *p* = 0.660 2-4 s Pre: 3.64 ± 2.46 Post: 5.27 ± 2.97 Δ 1.63, *p* = 0.310 > 4 s Pre: 1.27 ± 1.10 Post: 1.64 ± 1.21 Δ 0.37, *p* = 0.900	*p* = 0.010 *p* = 0.070^#^ *p* = 0.370^#^ *p* = 0.010
	In-Game Pressure Applied When Executing Skill (Count of times player with the ball had or did not have opponent within 3 m when executing skill)	Pressure Pre: 2.27 ± 1.74 Post: 2.55 ± 1.21 Δ 0.28, *p* = 1.000 No Pressure Pre: 8.36 ± 2.50 Post: 7.82 ± 2.89 Δ -0.54,* p* = 0.980	Pressure Pre: 3.27 ± 2.33 Post: 7.18 ± 5.00 Δ 3.91,* p* = 0.020 No Pressure Pre: 7.55 ± 4.74 Post: 2.91 ± 1.51 Δ -4.64, *p* = 0.010	*p* = 0.050 *p* = 0.040
	Locomotor Movement When Executing a Skill (Count of times player was stationary or running when executing skill)	Stationary Pre: 7.73 ± 2.76 Post: 4.64 ± 1.21 Δ -3.09, *p* = 0.280 Running Pre: 2.82 ± 2.14 Post: 5.73 ± 2.20 Δ 2.91,* p* = 0.010	Stationary Pre: 7.55 ± 4.99 Post: 8.00 ± 5.40 Δ 0.45, *p* = 0.990 Running Pre: 3.27 ± 2.10 Post: 2.09 ± 1.51 Δ -1.18,* p* = 0.520	*p* = 0.150 *p* = < 0.010
Cheong, et al. [34] (Hockey)	Dribble Test (sec.)	Pre: 5.01 ± 1.23 Post: 3.68 ± 0.77 Δ -1.33,* p* = N.S. ^a^	Pre: 4.92 ± 1.25 Post: 3.83 ± 0.71 Δ -1.09, *p* = N.S. ^a^	*p* = 0.720^#^
	Push Test- Accuracy (Scale 1–5)	Pre: 2.42 ± 1.10 Post: 3.16 ± 0.77 Δ 0.74,* p* = N.S. ^a^	Pre: 2.48 ± 0.93 Post: 3.34 ± 0.54 Δ 0.86, *p* = N.R	*p* = 0.210^#^
	Push Speed Test (km/h)	Pre: 31.93 ± 4.57 Post: 38.28 ± 5.90 Δ 6.35,* p* = N.S. ^a^	Pre: 33.90 ± 7.10 Post: 35.21 ± 7.06 Δ 1.31, *p* = N.S. ^a^	*p* = < 0.001
	In-Game % of Success- Trapping	Pre: 65.23 ± 9.82 Post: 82.73 ± 7.11 Δ 17.50, *p* = N.S. ^a^	Pre: 67.02 ± 13.59 Post: 74.68 ± 12.97 Δ 7.66,* p* = N.S. ^a^	*p* = 0.720^#^
	In-Game % of Success- Passing	Pre: 65.73 ± 15.25 Post: 72.27 ± 5.89 Δ 6.54, *p* = N.S. ^a^	Pre: 67.95 ± 15.98 Post: 69.47 ± 8.25 Δ 1.52,* p* = N.S. ^a^	*p* = 0.970^#^
	In-Game % of Success- Shooting	Pre: 79.17 ± 33.23 Post: 52.20 ± 33.42 Δ -26.97, *p* = N.S. ^a^	Pre: 65.00 ± 31.83 Post: 69.45 ± 18.76 Δ 4.45, *p* = N.S. ^a^	*p* = 0.130^#^
	In-Game % of Success- Dribbling	Pre: 86.48 ± 14.37 Post: 91.80 ± 4.42 Δ 5.32,* p* = N.S. ^a^	Pre: 92.23 ± 10.02 Post: 88.98 ± 7.44 Δ -3.25, *p* = N.S. ^a^	*p* = 0.820^#^
	In-Game Successful Trapping (Attempts)	Pre: 18.67 ± 7.71 Post: 22.17 ± 2.48 Δ 3.50, *p* = N.S. ^a^	Pre: 19.33 ± 9.56 Post: 20.83 ± 8.61 Δ 1.50, *p* = N.S. ^a^	*p* = N.S.^a^
	In-Game Successful Passing (Attempts)	Pre: 21.67 ± 5.92 Post: 22.83 ± 4.40 Δ 1.16, *p* = N.S. ^a^	Pre: 24.00 ± 9.84 Post: 22.67 ± 8.36 Δ -1.33, *p* = N.S. ^a^	*p* = N.S. ^a^
	In-Game Successful Shooting (Attempts)	Pre: 1.33 ± 0.52 Post: 2.67 ± 2.50 Δ 1.34,* p* = N.S. ^a^	Pre: 1.33 ± 0.82 Post: 2.50 ± 1.05 Δ 1.17,* p* = N.S. ^a^	*p* = 0.110^#^
	In-Game Successful Dribbling (Attempts)	Pre: 7.83 ± 3.19 Post: 14.67 ± 3.67 Δ 6.84,* p* = N.S. ^a^	Pre: 8.67 ± 4.80 Post: 11.17 ± 2.93 Δ 2.50, *p* = N.S. ^a^	*p* = 0.040
	In-Game Total Attempts- Trapping	Pre: 27.83 ± 7.94 Post: 26.83 ± 2.48 Δ -1.00, *p* = N.S. ^a^	Pre: 28.00 ± 10.08 Post: 27.50 ± 8.07 Δ -0.50,* p* = N.S. ^a^	*p* = N.S. ^a^
	In-Game Total Attempts- Passing	Pre: 32.83 ± 5.00 Post: 31.50 ± 4.97 Δ -1.33, *p* = N.S. ^a^	Pre: 34.67 ± 8.66 Post: 32.00 ± 7.69 Δ -2.67,* p* = N.S. ^a^	*p* = N.S. ^a^
	In-Game Total Attempts- Shooting	Pre: 2.00 ± 1.10 Post: 5.17 ± 3.37 Δ 3.17, *p* = N.S. ^a^	Pre: 2.00 ± 1.67 Post: 3.67 ± 1.37 Δ 1.67, *p* = N.S. ^a^	*p* = 0.350^#^
	In-Game Total Attempts- Dribbling	Pre: 9.00 ± 3.03 Post: 15.67 ± 3.67 Δ 6.67, *p* = N.S. ^a^	Pre: 8.83 ± 4.58 Post: 12.83 ± 4.07 Δ 4.00,* p* = N.S. ^a^	*p* = 0.400^#^
Chow, et al. [38] (Soccer)	In-Game Pass Execution (Count of Successful Attempt)	Pre: 8.50 ± 4.77 Post: 13.21 ± 5.28 Δ 4.71, *p* = 0.003	Pre: 8.64^#^ ± 5.05^#^ Post: 10.95^#^ ± 4.46^#^ Δ 2.31, *p* = 0.771^#^	*p* = 0.117^#^^
	Types of Passes (Count of Different Types of Passes Executed)	Pre: 5.86 ± 2.37 Post: 9.18 ± 3.62 Δ 3.32, *p* = < 0.001	Pre: 6.15^#^ ± 3.68^#^ Post: 6.65^#^ ± 2.72^#^ Δ 0.50,* p* = 1.000^#^	*p* = 0.005^
	Possession of Ball (sec)	Pre: 63,114 ± 27,342 Post: 87,693 ± 27,410 Δ 24,579, *p* = 0.010	Pre: 57,184 ± 24,105 Post: 90,642 ± 32,697 Δ 33,458, *p* = 0.001	*p* = 0.720^#^^
	Consecutive Passes Made (Count)	Pre: 2.54^#^ ± 2.89^#^ Post: 4.32^#^ ± 2.64^#^ Δ 1.78,* p* = 0.161^#^	Pre: 3.65^#^ ± 3.31^#^ Post: 3.35^#^ ± 2.82^#^ Δ -0.30, *p* = 1.000^#^	*p* = 0.269^#^^
	Goals Scored (Count)	Pre: 1.46^#^ ± 1.99^#^ Post: 2.79^#^ ± 2.01^#^ Δ 1.33, *p* = 0.072^#^	Pre: 1.59^#^ ± 1.92^#^ Post: 2.91^#^ ± 2.07^#^ Δ 1.32, *p* = 0.156^#^	*p* = 0.824^#^^
	Possession Turnovers (Count)	Pre: 17.00 ± 7.54 Post: 26.13 ± 1.85 Δ 9.13, *p* = 0.048	Pre: 11.08 ± 7.23 Post: 18.83 ± 11.22 Δ 7.75,* p* = 0.257	*p* = 0.044^#^^
Deuker, et al. [39] (Soccer)	Passing Test (sec.)	Pre: 31.03 ± 3.91 Post: 28.93 ± 3.25 Δ -2.10, *p* = 0.002	Pre: 31.39 ± 2.59 Post: 27.76 ± 2.76 Δ -3.63, *p* = < 0.001	*p* = 0.554^#^
	Dribbling Test (sec.)	Pre: 14.45 ± 2.02 Post: 14.25 ± 1.84 Δ -0.20,* p* = N.S. ^a^	Pre: 13.32 ± 1.44 Post: 13.21 ± 1.16 Δ -0.11,* p* = N.S. ^a^	*p* = 0.146^#^
Esposito, et al. [42] (Soccer)	Technical Passing (Loughborough Soccer Passing Test)	Pre: 59.90 ± 0.50 Post: 50.30 ± 0.60 Δ -9.60,* p* = 0.001	Pre: 60.20 ± 0.60 Post: 56.30 ± 0.60 Δ -3.90,* p* = 0.001	*p* = 0.001
Mohammadi Orangi, et al. [40] (Soccer)	In-Game Actions (Count of Skills Executed per player)	Pre: No pre-intervention Post: 74.70 ± 8.20	Pre: No pre-intervention Post: 65.20 ± 10.70	*p* = 0.010
	In-Game Adequate Actions (Count of Skills Executed per player) (% of total actions)	Pre: No pre-intervention Post: 40.20 ± 8.30 (53.8%)	Pre: No pre-intervention Post: 32.70 ± 9.30 (50.1%)	*p* = 0.090
	Variability of In-Game Actions (Count of Skills Executed per player) (% of total actions)	Pre: No pre-intervention Post: 19.30 ± 5.3 (25.8%)	Pre: No pre-intervention Post: 13.10 ± 5.80 (19.98%)	*p* = 0.010
	In-Game Original Actions (Count of Skills Executed per player) (% of total actions)	Pre: No pre-intervention Post: 1.14 ± 0.77 (1.5%)	Pre: No pre-intervention Post: 0.45 ± 0.51 (0.6%)	*p* = < 0.001
	In-Game Creative Actions (Count of Skills Executed per player) (% of total actions)	Pre: No pre-intervention Post: 0.45 ± 0.67 (0.6%)	Pre: No pre-intervention Post: 0.14 ± 0.35 (0.2%)	*p* = < 0.001
Praxedes, et al. [36] (Soccer)	In-Game Pass Execution (0–1 Rating)	Pre: N.S Post: 0.714 ± 0.052	Pre: N.S Post: 0.558 ± 0.133	*p* = 0.003
	In-Game Dribbling Execution (0–1 Rating)	Pre: N.S Post: 0.807 ± 0.092	Pre: N.S Post: 0.715 ± 0.161	*p* = 0.143
	In-Game Decision Making- Passing (0–1 Rating)	Pre: N.S Post: 0.843 ± 0.039	Pre: N.S Post: 0.661 ± 0.111	*p* = < 0.001
	In-Game Decision Making- Dribbling (0–1 Rating)	Pre: N.S Post: 0.786 ± 0.114	Pre: N.S Post: 0.732 ± 0.156	*p* = 0.486
Roberts, et al. [41] (Soccer)	Strong Foot Finishing (Loughborough Shooting Skills Test)	Pre: 25.06 ± 3.46 Post: 27.02 ± 3.40 Δ 1.96, *p* = 0.210	Pre: 26.34 ± 2.62 Post: 26.46 ± 3.40 Δ 0.12, *p* = 0.260	*p* = 0.190
	Weak Foot Finishing (Loughborough Shooting Skills Test)	Pre: 14.56 ± 5.67 Post: 16.56 ± 5.67 Δ 2.00, *p* = 0.680	Pre: 15.01 ± 4.89 Post: 16.01 ± 4.89 Δ 1.00, *p* = 0.290	*p* = 0.350
	1 v 1 Attack (% of Success)	Pre: 68.56 ± 14.21 Post: 78.56 ± 14.21 Δ 10.00, *p* = 0.050	Pre: 65.22 ± 14.47 Post: 68.56 ± 14.21 Δ 3.34,* p* = 0.790	*p* = 0.020
	Decision Making (% of Correct Executions)	Pre: 64.25 ± 16.67 Post: 81.25 ± 16.67 Δ 17.00, *p* = 0.010	Pre: 60.33 ± 12.08 Post: 68.25 ± 16.67 Δ 7.92,* p* = 0.620	*p* = 0.010

N.S. = Not Significant and no *p-value* reported

N.S.^a^ = Not Significant and confirmed with author

^#^ = Data provided by author upon request

^ = *p-value* calculated at post-intervention test timepoint

The descriptive synthesis examined whether there was a significant interaction effect favouring nonlinear or linear pedagogy for each outcome in the development of technical skills (Table 4) or tactical skills (Table 5). Records that did not provide a *p*-value or reported a *p-*value > 0.05 for an outcome were recorded as *‘No significant difference reported between groups’*. Over one-third (34%) of outcomes documented across nine studies favoured nonlinear pedagogical approaches to technical skill development, with most (66%) indicating no significant difference between pedagogical approaches (Table 4). Consequently, the descriptive synthesis summary effect rating (0) suggested that nonlinear approaches to technical skills development were at least equally effective as linear pedagogical approaches and the removal of studies with serious risk of bias did not alter the summary effect score. Well over half (62.5%) of outcomes documented across four studies favoured nonlinear pedagogical approaches to tactical skill development, with only one outcome favouring linear pedagogy (Table 5). With all four studies included in the descriptive synthesis, the summary effect rating suggested the results were inconclusive (?) as the threshold of 66% was not reached. However, when the study with serious risk of bias [36] was removed from the descriptive synthesis in a sensitivity analysis, the summary effect rating changed to ( +), suggesting nonlinear pedagogy was more effective than linear pedagogy for developing tactical skills in team-invasion ball sports.

**Table 4:** Descriptive synthesis of studies exploring the effects of nonlinear (NLP) compared to linear (LP) pedagogy for technical skill outcomes

Sport	Intervention Outcomes		% of Outcomes Favouring NLP	Summary Effect
	Significant Difference Reported Between Groups (Favouring NLP)	No Significant Difference Reported Between Groups		
Australian Rules Football	• Bonney, et al. [37] (n = 22) o Kick Distance: Effect Only Found for Kicks 0-20 m and 20-40 m o Kick Locomotor Movement: Effect Only Found When on The Run and Not Stationary o Kick Proficiency		3 / 3 = 100%	( +)^
Hockey	• Cheong, et al. [34] (n = 48) o In-Game Successful Dribbling o Push Speed Test	• Cheong, et al. [34] (n = 48) o Hockey Dribble Test o In-Game % of Success—Trapping o In-Game % of Success—Passing o In-Game % of Success—Shooting o In-Game % of Success—Dribbling o In-Game Successful Trapping o In-Game Successful Passing o In-Game Successful Shooting o In-Game Total Attempts—Trapping o In-Game Total Attempts—Passing o In-Game Total Attempts—Shooting o In-Game Total Attempts—Dribbling o Push Test- Accuracy	2 / 15 = 13%	( 0)^
Soccer	• Chow, et al. [38] (n = 224) o Types of Passes • Esposito, et al., [42] (n = 30) o Technical Passing • Mohammadi Orangi, et al. [40] (n = 64) o In-Game Actions o Variability of In-Game Actions o In-Game Original Actions[Skill Action Performed by 3 or Less Players] o In-Game Creative Actions[Skill Action Original AND Adequate] • Praxedes, et al. [36] (n = 19) o In-Game Pass Execution	• Abate Daga, et al. [35] (n = 31) o Shuttle Dribble Test • Chow, et al. [38] (n = 224) o In-Game Pass Execution o Consecutive Passes Made o Goals Scored • Deuker, et al. [39] (n = 28) o Dribbling Test o Passing Test • Mohammadi Orangi, et al. [40] (n = 64) o In-Game Adequate Actions[Skill Action Progressed Game] • Praxedes, et al. [36] (n = 19) o In-Game Dribbling • Roberts, et al. [41] (n = 22) o Strong-Foot Finishing o Weak-Foot Finishing	7 / 17 = 41%	( ?)
TOTAL			12 / 35 = 34% 11 / 32 = 34%^#^	( 0) ( 0)^#^

Significant Difference Favouring NLP: Group x Time OR Group (if no pre-intervention assessment) *p-value* < 0.05

^ Results influenced by a single study only

No Significant Difference Reported Between Groups: Group x Time OR Group (if no pre-intervention assessment) *p-value* > 0.05 / no *p-value* reported

Summary Effect: + = ≥ 66% of outcomes significantly favouring NLP; 0 = ≥ 66% of outcomes demonstrated no significant difference between NLP and LP; ? = Threshold of 66% of outcomes was not reached by NLP or LP

^#^ Denotes findings are the result of a sensitivity analysis where studies with serious/high risk of bias are removed from the synthesis

- Serious/High risk of bias; - Some concerns with bias / moderate risk of bias; - Low risk of bias

**Table 5:** Descriptive synthesis of studies exploring the effects of nonlinear (NLP) compared to linear (LP) pedagogy for tactical skill outcomes

Sport	Intervention Outcomes			% of Outcomes Favouring NLP	Summary Effect
	Significant Difference Reported Between Groups (Favouring NLP)	No Significant Difference Reported Between Groups	Significant Difference Reported Between Groups (Favouring LP)		
Australian Rules Football	• Bonney, et al. [37] (n = 22) o Time with Ball Before Skill Execution: Effect Only Found for < 1 s. and > 4 s o Pressure Applied When Executing Skill			2 / 2 = 100%	( +)^
Soccer	• Praxedes, et al. [36] (n = 19) o Decision Making- Passing • Roberts, et al. [41] (n = 22) o 1 v 1 Attack o Decision Making	o Chow, et al. [38] (n = 224) Possession of Ball • Praxedes, et al. [36] (n = 19) Decision Making- Dribbling	• Chow, et al. [38] (n = 224) Possession Turnovers	3 / 6 = 50%	( 0)
TOTAL				5 / 8 = 62.5% 4 / 6 = 66%^#^	( ?) ( +)^#^

Significant Difference Favouring NLP: Group x Time OR Group (if no pre-intervention assessment) *p-value* < 0.05. ^ Results influenced by a single study only

No Significant Difference Reported Between Groups: Group x Time OR Group (if no pre-intervention assessment) *p-value* > 0.05 / no *p-value* listed

Summary Effect: + = ≥ 66% of outcomes significantly favouring NLP; 0 = ≥ 66% of outcomes demonstrated no significant difference between NLP and LP; ? = Threshold of 66% of outcomes was not reached by NLP or LP

^#^ Denotes findings are the result of a sensitivity analysis where studies with serious/high risk of bias are removed from the synthesis

- Serious/High risk of bias; - Some concerns with bias / moderate risk of bias; - Low risk of bias

## Discussion

The aim of this systematic review was to critically evaluate literature that compared the relative effectiveness of linear and nonlinear pedagogies for supporting technical and tactical skill development in team-invasion ball sports. For many of the outcomes, both the linear and nonlinear groups significantly improved from pre- to post-intervention testing. Whilst over a third (34%) of outcomes were shown to favour a nonlinear approach to technical skill development, and none favoured linear, our descriptive synthesis revealed that overall, the majority of research demonstrated no statistically significant difference between linear and nonlinear approaches for developing technical skill outcomes in team-invasion ball sports. However, our descriptive synthesis did reveal that nonlinear approaches were more effective, overall, than linear approaches for developing tactical skills in team-invasion ball sports. These results are important for coaches and learning design specialists, as they suggest that nonlinear pedagogical approaches may be more effective in supporting tactical and, to a lesser extent, technical skill development when compared to linear pedagogical approaches in team-invasion ball sports. Our results are somewhat consistent with those of Chow et al. [18], who proposed that nonlinear pedagogy facilitates skill development and may offer some advantages over a linear pedagogy. These results would also appear to support some of the suggestions put forward by Chow [17], Davids et al. [43], Renshaw et al. [44] and Woods et al. [15], who each propose the beneficial nature of using a nonlinear pedagogical approach to support skill development in various sporting contexts.

Regarding tactical skill outcomes, five from a total of eight outcomes had a significant difference favouring nonlinear pedagogy, while a single outcome favoured linear pedagogy. A sensitivity analysis, removing studies with serious/high risk of bias, further strengthened the results favouring nonlinear pedagogy in 66% of outcomes. Nonlinear pedagogy encourages performers to search, discover and explore solutions to various movement problems [16]. Therefore, if implemented coherently, a nonlinear pedagogical approach has the potential to elicit greater improvement in tactical outcomes due to the nature of performers continually adapting their movement in response to changes in environing conditions [21]. Relevant to coaches and practitioners of team-invasion ball sport athletes, this finding suggests that a practice environment should utilise sample constraints from competition to ensure the *representativeness* of tasks is maintained. By representing competitive environments in practice, nonlinear pedagogies have the potential to lead to greater tactical skill development relative to linear pedagogical approaches that prioritise a ‘correct’ or ‘idealised’ way of doing.

When developing technical skills, the studies showed that 34% of the reported outcomes favoured a nonlinear pedagogical approach, while the remaining 66% showed no statistical difference between linear and nonlinear. Bearing in mind that there were no outcomes which favoured linear pedagogical approaches directly, it is evident that both approaches produce positive results when learning technical skills in team-invasion ball sports. Even when applying a sensitivity analysis, the descriptive synthesis does not alter the summary effect, showing that the majority of outcomes (66%) reported no significant difference between groups. Further research that directly compares linear and nonlinear pedagogy in team-invasion ball sports is necessary, as our findings are based on a relatively small number of studies and, in many cases, small sample sizes. Along these lines, we would also suggest that future research could aim to quantify the relative effectiveness of linear and nonlinear pedagogical approaches within populations of differing developmental levels.

A unique observation from this review was that over half of the studies included appeared to use assessments of ‘skill’ that were somewhat removed from the key demands of competition. An example of this was by Cheong et al. [34] who implemented a Hockey Dribbling Test. Notably, this test required participants to dribble a ball using their stick as fast as possible through a course that included cones and agility poles rather than other players. Though this is a repeatable assessment instrument, the environment was overly stable and arguably, non-representative of the constraints of competition. This observation, to us, speaks to an inherent lack of representativeness in the assessment of skill across the literature analysed, irrespective of the pedagogical intervention used. This is important to consider as assessments of skill that do not adequately represent the demands of competition may dilute the effects of the subsequent pedagogical intervention utilised and question the validity of many of these skill assessments used in the literature. According to Nathan et al. [45], a lack of representativeness in assessment may situate learning as unfolding in contexts that do not meet the demands of competition. With this in mind, it is our recommendation that to better determine the effectiveness of a pedagogical intervention, the assessment task must possess high levels of both task functionality and action fidelity to ensure they represent the demands encountered in competition [46]. This presents an important, albeit unintended, consequence of our review that should guide future studies that intend to determine the effectiveness of various pedagogical approaches in the sport sciences.

Relative to the sporting context, there was a significant number of studies representing a soccer environment, accounting for 78% of included results. This is a limiting factor regarding the global representation of team-invasion ball sports, where the results could be determined as being skewed to one sport alone. As a more comprehensive picture, the studies encompassed in this review are only representative of three sports, with Australian Rules Football and field hockey being the other two. Therefore, an important recommendation for future research is to explore a wider range of team-invasion ball sports to offer a more comprehensive insight into the dynamics of skill development in a variety of performance environments.

Across the studies included in this review, there was a mean of 15.8 ± 6.6 sessions across 7.00 ± 3.2 weeks, akin to approximately two sessions per week. When exploring intervention durations, a short-term (≤ 6 sessions), mid-term (> 6 but ≤ 24 sessions), or long-term (≥ 25 sessions) approach can be implemented, as used in a review by Bergmann et al. [23]. Using Bergmann et al.’s methodology, the studies included here could be described as representing one short-term, seven mid-term and no long-term interventions. Whether a focused longitudinal study is required is an interesting point to consider, as skill development appears to occur nonlinearly, across different contexts and timescales [47, 48]. Given this, it would make it challenging for both researchers and practitioners to concretely state how long an intervention should be maintained for optimal and retained outcomes. Nevertheless, as linear and nonlinear pedagogies were typically both effective for improving a range of technical and tactical outcomes, greater duration interventions and longer-term follow-up may be required to better demonstrate whether one approach is more effective than the other.

## Conclusion

This systematic review explored the effectiveness of linear and nonlinear pedagogical approaches in team-invasion ball sports for the development of technical and tactical skills. It was found that nonlinear pedagogy led to greater positive effects relative to linear pedagogy for developing tactical skills. Regarding technical skill development, several outcomes had a significant difference favouring nonlinear pedagogy, while no outcomes favoured linear pedagogy. Though this is the case, the majority of literature shows that there are no significant differences between approaches when developing technical skills. While not an intended outcome, it was found that further analysis into the representativeness of assessment instruments is an important takeaway for researchers interested in measuring the effectiveness of a certain pedagogical intervention. Importantly, continued research is required to further explore these findings across a wider range of contexts with larger participant populations, while also examining the length of studies for effective skill development to take place.

## Supplementary Information

Additional file 1.

Additional file 2.

## Data Availability

All available data not included in the manuscript can be found in the Supplementary Information (SI) Files.

## Abbreviations

LP: Linear pedagogy
NLP: Nonlinear pedagogy
N.S.: Not significant
PICO: Population, intervention, comparison/control, outcome
PRISMA: Preferred reporting items for systematic reviews and meta-analyses
RoB 2: Risk of bias 2
ROBINS-I: Risk of bias in non-randomized studies–interventions
Robvis: Risk of bias visualisation

## References

[1] Woods CT, McKeown I, O’Sullivan M, Robertson S, Davids K. Theory to practice: PERFORMANCE preparation models in contemporary high-level sport guided by an ecological dynamics framework. Sports Med Open. 2020;6(1):36. 10.1186/s40798-020-00268-5. 32797290 10.1186/s40798-020-00268-5 PMC7427670

[2] Woods CT, Araujo D, Davids K. On finding one's way: a comment on Bock et al. (2024). Psychol Res. 2024;88(7):2172–9. 10.1007/s00426-024-02011-1. 10.1007/s00426-024-02011-1 PMC11449947 39052102

[3] Heft H. Ecological psychology in context. Resources for ecological psychology. Mahwah, N.J: L. Erlbaum Associates; 2001.

[4] Heft H. Wayfinding, navigation, and environmental cognition from a naturalist's stance. Handbook of spatial cognition. Washington, DC: American Psychological Association; 2013. p. 265–94.

[5] Di Tore PA, Schiavo R, D’Isanto T. Physical education, motor control and motor learning: Theoretical paradigms and teaching practices from kindergarten to high school. J Phys Educ Sp. 2016;16(4):1293–7. 10.7752/jpes.2016.04205.

[6] Fitts P, Posner M. Human performance. Basic concepts in psychology series. Belmont, Calif: Brooks Cole; 1967.

[7] van der Kamp J, Withagen R, Orth D. On the education about/of radical embodied cognition. Front Psychol. 2019. 10.3389/fpsyg.2019.02378. 10.3389/fpsyg.2019.02378 PMC6848271 31749732

[8] Araujo D, Davids K. What exactly is acquired during skill acquisition? J Conscious Stud. 2011;18(3):7–23.

[9] Davids K, Araujo D. The concept of “Organismic Asymmetry” in sport science. J Sci Med Sport. 2010;13(6):633–40. 10.1016/j.jsams.2010.05.002. 20580313 10.1016/j.jsams.2010.05.002

[10] Button C, Seifert L, Chow JY, Araújo D, Davids K. Dynamics of skill acquisition: an ecological dynamics approach. Second edition. ed. Champaign, IL: Human Kinetics; 2021.

[11] Corbetta D, Vereijken B. Understanding development and learning of motor coordination in sport: The contribution of dynamic systems theory. Int J Sport Psychol. 1999;30(4):507–30.

[12] Woods CT, Rudd J, Gray R, Davids K. Enskilment: an ecological-anthropological worldview of skill, learning and education in sport. Sports Med Open. 2021;7(1):33. 10.1186/s40798-021-00326-6. 34019196 10.1186/s40798-021-00326-6 PMC8140044

[13] Standing R, Maulder P. The effectiveness of progressive and traditional coaching strategies to improve sprint and jump performance across varying levels of maturation within a general youth population. Sports. 2019;7(8):186. 10.3390/sports7080186. 31366104 10.3390/sports7080186 PMC6723898

[14] Crotti M, Rudd JR, Roberts S, Boddy LM, Fitton Davies K, O’Callaghan L, et al. Effect of linear and nonlinear pedagogy physical education interventions on children’s physical activity: A cluster randomized controlled trial (SAMPLE-PE). Children. 2021. 10.3390/children8010049. 10.3390/children8010049 PMC7830495 33467568

[15] Woods CT, McKeown I, Rothwell M, Araújo D, Robertson S, Davids K. Sport practitioners as sport ecology designers: How ecological dynamics has progressively changed perceptions of skill “acquisition” in the sporting habitat. Front Psychol. 2020;11:654. 10.3389/fpsyg.2020.00654. 32390904 10.3389/fpsyg.2020.00654 PMC7194200

[16] Rudd J, Renshaw I, Savelsbergh G, Chow JY, Roberts W, Newcombe D, et al. Nonlinear pedagogy and the athletic skills model : The importance of play in supporting physical literacy. Milton: Taylor & Francis Group; 2021.

[17] Chow JY. Nonlinear learning underpinning pedagogy: Evidence, challenges, and implications. Quest. 2013;65(4):469–84. 10.1080/00336297.2013.807746.

[18] Chow JY, Davids K, Button C, Renshaw I. Nonlinear pedagogy in skill acquisition: an introduction. 2nd ed. New York, NY: Routledge; 2022.

[19] Spittle M. Motor learning and skill acquisition : applications for physical education and sport. 2nd ed. London: Macmillan Education UK; 2021.

[20] Kolman NS, Kramer T, Elferink-Gemser MT, Huijgen BCH, Visscher C. Technical and tactical skills related to performance levels in tennis: a systematic review. J Sports Sci. 2019;37(1):108–21. 10.1080/02640414.2018.1483699. 29889615 10.1080/02640414.2018.1483699

[21] Davids K. Learning design for nonlinear dynamical movement systems. Open Sports Sci J. 2012;5(1):9–16. 10.2174/1875399X01205010009.

[22] Lowe GC, Owen JA, Gottwald VM, Jones ES. Design, validation, and reliability of the Bangor rugby assessment tool for evaluating technical and tactical skills in rugby union development pathways. Front Sports Act Living. 2025. 10.3389/fspor.2025.1568302. 10.3389/fspor.2025.1568302 PMC12055826 40336709

[23] Bergmann F, Gray R, Wachsmuth S, Höner O. Perceptual-motor and perceptual-cognitive skill acquisition in soccer: a systematic review on the influence of practice design and coaching behavior. Front Psychol. 2021;12:772201. 10.3389/fpsyg.2021.772201. 34925173 10.3389/fpsyg.2021.772201 PMC8675907

[24] Clark ME, McEwan K, Christie CJ. The effectiveness of constraints-led training on skill development in interceptive sports: a systematic review. Int J Sports Sci Coach. 2019;14(2):229–40. 10.1177/1747954118812461.

[25] Page MJ, McKenzie JE, Bossuyt PM, Boutron I, Hoffmann TC, Mulrow CD, et al. The PRISMA 2020 statement: an updated guideline for reporting systematic reviews. BMJ. 2021;372: n71. 10.1136/bmj.n71. 33782057 10.1136/bmj.n71 PMC8005924

[26] Clark JM, Sanders S, Carter M, Honeyman D, Cleo G, Auld Y, et al. Improving the translation of search strategies using the Polyglot Search Translator: a randomized controlled trial. J Med Libr Assoc. 2020;108(2):195–207. 10.5195/jmla.2020.834. 32256231 10.5195/jmla.2020.834 PMC7069833

[27] Coleman K, Norris S, Weston A, Grimmer-Somers K, Hillier S, Merlin T, et al. NHMRC additional levels of evidence and grades for recommendations for developers of guidelines. In: National Health and Medical Research Council, editor.: Australian Government; 2009.

[28] Sterne JA, Hernan MA, Reeves BC, Savovic J, Berkman ND, Viswanathan M, et al. ROBINS-I: a tool for assessing risk of bias in non-randomised studies of interventions. BMJ. 2016;355: i4919. 10.1136/bmj.i4919. 27733354 10.1136/bmj.i4919 PMC5062054

[29] Sterne JAC, Savovic J, Page MJ, Elbers RG, Blencowe NS, Boutron I, et al. RoB 2: a revised tool for assessing risk of bias in randomised trials. BMJ. 2019;366: l4898. 10.1136/bmj.l4898. 31462531 10.1136/bmj.l4898

[30] McGuinness LA, Higgins JPT. Risk-of-bias VISualization (robvis): an R package and Shiny web app for visualizing risk-of-bias assessments. Res Synth Methods. 2021;12(1):55–61. 10.1002/jrsm.1411. 32336025 10.1002/jrsm.1411

[31] Deeks JJ, Higgins JPT, Altman DG. Analysing data and undertaking meta‐analyses. Cochrane Handbook for Systematic Reviews of Interventions. Chichester, UK: John Wiley & Sons, Ltd; 2019. p. 241–84.

[32] Milne N, Miao M, Beattie E. The effects of serial casting on lower limb function for children with Cerebral Palsy: a systematic review with meta-analysis. BMC Pediatr. 2020;20(1):324. 10.1186/s12887-020-02122-9. 32615954 10.1186/s12887-020-02122-9 PMC7330971

[33] The EndNote Team. EndNote. EndNote 20 ed. Philadelphia, PA: Clarivate; 2013.

[34] Cheong JP, Lay B, Razman R. Investigating the contextual interference effect using combination sports skills in open and closed skill environments. J Sports Sci Med. 2016;15(1):167–75. 26957940 PMC4763836

[35] Abate Daga F, Baseggio L, Gollin M, Beratto L. Game-based versus multilateral approach: effects of a 12-week program on motor skill acquisition and physical fitness development in soccer school children. J Sports Med Phys Fitness. 2020;60(9):1185–93. 10.23736/S0022-4707.20.10726-6. 32432448 10.23736/S0022-4707.20.10726-6

[36] Praxedes A, Del Villar F, Pizarro D, Moreno A. The impact of nonlinear pedagogy on decision-making and execution in youth soccer players according to game actions. J Hum Kinet. 2018;62:185–98. 10.1515/hukin-2017-0169. 29922390 10.1515/hukin-2017-0169 PMC6006529

[37] Bonney N, Larkin P, Ball K. Kick proficiency and skill adaptability increase from an Australian football small-sided game intervention. Front Sports Act Living. 2022;4:1026935. 10.3389/fspor.2022.1026935. 36385779 10.3389/fspor.2022.1026935 PMC9643701

[38] Chow JY, Meerhoff LA, Choo CZY, Button C, Tan BS. The effect of nonlinear pedagogy on the acquisition of game skills in a territorial game. Front Psychol. 2023;14:1077065. 10.3389/fpsyg.2023.1077065. 36814665 10.3389/fpsyg.2023.1077065 PMC9940013

[39] Deuker A, Braunstein B, Chow JY, Fichtl M, Kim H, Körner S, et al. “Train as you play”: Improving effectiveness of training in youth soccer players. Int J Sports Sci Coach. 2023;19(2):677–86. 10.1177/17479541231172702.

[40] Mohammadi Orangi B, Yaali R, Bahram A, van der Kamp J, Aghdasi MT. The effects of linear, nonlinear, and differential motor learning methods on the emergence of creative action in individual soccer players. Psychol Sport Exerc. 2021;56:N.PAG-N.PAG.

[41] Roberts S, Rudd J, Reeves M. Efficacy of using non-linear pedagogy to support attacking players’ individual learning objectives in elite-youth football: A randomised cross-over trial. J Sports Sci. 2020;38(11–12):1454–64. 10.1080/02640414.2019.1609894. 31030644 10.1080/02640414.2019.1609894

[42] Esposito G, Ceruso R, Aliberti S, Raiola G. Ecological-dynamic approach vs. traditional prescriptive approach in improving technical skills of young soccer players. J Funct Morphol Kinesiol. 2024;9(3):162. 10.3390/jfmk9030162. 39311270 10.3390/jfmk9030162 PMC11417948

[43] Davids K, Araujo D, Correia V, Vilar L. How small-sided and conditioned games enhance acquisition of movement and decision-making skills. Exerc Sport Sci Rev. 2013;41(3):154–61. 10.1097/JES.0b013e318292f3ec. 23558693 10.1097/JES.0b013e318292f3ec

[44] Renshaw I, Chow J, Davids K, Hammond J. A constraints-led perspective to understanding skill acquisition and game play: a basis for integration of motor learning theory and physical education praxis? Phys Educ Sport Pedagogy. 2010;15(2):117–37.

[45] Nathan S, Salimin N, Shahril MI. A comparative analysis of badminton game instructions effect of non-linear pedagogy and linear pedagogy. J Appl Fundam Sci. 2018;9(6S):1258. 10.4314/jfas.v9i6s.94.

[46] Pinder RA, Renshaw I, Davids K. The role of representative design in talent development: a comment on “Talent identification and promotion programmes of Olympic athletes.” J Sports Sci. 2013;31(8):803–6. 10.1080/02640414.2012.718090. 22943131 10.1080/02640414.2012.718090

[47] Chow JY, Komar J, Seifert L. The role of nonlinear pedagogy in supporting the design of modified games in junior sports. Front Psychol. 2021. 10.3389/fpsyg.2021.744814. 10.3389/fpsyg.2021.744814 PMC8586551 34777136

[48] Vaughan J, Mallett CJ, Potrac P, Woods C, O’Sullivan M, Davids K. Social and cultural constraints on football player development in stockholm: Influencing skill, learning, and wellbeing. Front Sports Act Living. 2022;4:832111. 10.3389/fspor.2022.832111. 35669555 10.3389/fspor.2022.832111 PMC9163368

