# Supplementary material for: The Effectiveness of Linear and Nonlinear Pedagogical Approaches in Team-Invasion Ball Sports: A Systematic Review
Source: Sports Med Open. 2025 Aug 4;11:90. doi: 10.1186/s40798-025-00893-y (PMC12321719; doi:10.1186/s40798-025-00893-y)
Supplement: Supplementary file 1 — Additional file 1. [file 40798_2025_893_MOESM1_ESM.docx]

**Supplementary Information File 1- Search Strategies**

The effectiveness of linear and nonlinear pedagogical approaches in team-invasion ball sports: A systematic review

Journal of Sports Medicine

Liam Bromilow^1^, Nikki Milne^1^, Carl T. Woods^2,3^, Caroline Dowsett^1^ and Justin W. L Keogh^1,4,5^

^1^ Faculty of Health Sciences and Medicine, Bond University, Gold Coast, Australia

^2^ Institute for Health and Sport, Victoria University, Melbourne, Australia

^3^ Sport and Exercise Science, College of Healthcare Sciences, James Cook University

^4^ Sports Performance Research Centre New Zealand, Auckland University of Technology, Auckland, New Zealand

^5^ Kasturba Medical College, Mangalore, Manipal Academy of Higher Education, Manipal, Karnataka, India

Correspondence

Liam Bromilow: [liam.bromilow@student.bond.edu.au](mailto:liam.bromilow@student.bond.edu.au); ORCID: 0009-0007-7643-8454

Nikki Milne: 0000-0002-5121-9825

Carl Woods: 0000-0002-7129-8938

Caroline Dowsett: 0000-0001-7734-9436

Justin Keogh: 0000-0001-9851-1068

Supplementary Information File 1 - Search Strategies

| **Database**  **(Results)** | **Search Terms** |
| --- | --- |
| EmBase | (Rugby*:ti,ab OR Soccer:ti,ab OR Football:ti,ab OR oztag:ti,ab OR futsal:ti,ab OR lacrosse:ti,ab OR hockey:ti,ab OR hurling:ti,ab OR 'Water Polo':ti,ab OR Basketball:ti,ab OR Netball:ti,ab OR Handball:ti,ab OR Rugby/exp OR Soccer/exp OR Football/exp OR Hockey/exp OR Basketball/exp)  AND  (coach*:ti,ab OR teach*:ti,ab OR mentor:ti,ab OR train*:ti,ab OR learn*:ti,ab OR player:ti,ab OR Teaching/exp OR Mentors/exp OR Learning/exp OR 'Physical Education and Training'/exp OR athletes/exp)  AND  ('non-linear pedagog*':ti,ab OR 'non linear pedagog*':ti,ab OR 'nonlinear pedagog*':ti,ab OR 'constraints-led approach':ti,ab OR 'constraints led approach':ti,ab OR 'indirect instruct*':ti,ab OR 'nonlinear dynamic':ti,ab OR 'non-linear dynamic':ti,ab OR 'linear pedagog*':ti,ab OR 'direct instruct*':ti,ab OR linear:ti,ab OR nonlinear:ti,ab OR traditional:ti,ab)  AND  (skill*:ti,ab OR competence:ti,ab OR capability:ti,ab OR ability:ti,ab OR perform*:ti,ab OR develop*:ti,ab OR precision:ti,ab OR proficiency:ti,ab OR technical:ti,ab OR tactical:ti,ab OR mechanism:ti,ab OR motivation:ti,ab OR prepar*:ti,ab OR readiness:ti,ab OR 'Motor Skills'/exp OR 'Athletic Performance'/exp OR Motivation/exp) |
| PubMed | (Rugby*[tiab] OR Soccer[tiab] OR Football[tiab] OR oztag[tiab] OR futsal[tiab] OR lacrosse[tiab] OR hockey[tiab] OR hurling[tiab] OR "Water Polo"[tiab] OR Basketball[tiab] OR Netball[tiab] OR Handball[tiab] OR Rugby[Mesh] OR Soccer[Mesh] OR Football[Mesh] OR Hockey[Mesh] OR Basketball[Mesh])  AND  (coach*[tiab] OR teach*[tiab] OR mentor[tiab] OR train*[tiab] OR learn*[tiab] OR player[tiab] OR Teaching[Mesh] OR Mentors[Mesh] OR Learning[Mesh] OR "Physical Education and Training"[Mesh] OR athletes[Mesh])  AND  ("non-linear pedagog*"[tiab] OR "non linear pedagog*"[tiab] OR "nonlinear pedagog*"[tiab] OR "constraints-led approach"[tiab] OR "constraints led approach"[tiab] OR "indirect instruct*"[tiab] OR "nonlinear dynamic"[tiab] OR "non-linear dynamic"[tiab] OR "linear pedagog*"[tiab] OR "direct instruct*"[tiab] OR linear[tiab] OR nonlinear[tiab] OR traditional[tiab])  AND  (skill*[tiab] OR competence[tiab] OR capability[tiab] OR ability[tiab] OR perform*[tiab] OR develop*[tiab] OR precision[tiab] OR proficiency[tiab] OR technical[tiab] OR tactical[tiab] OR mechanism[tiab] OR motivation[tiab] OR prepar*[tiab] OR readiness[tiab] OR "Motor Skills"[Mesh] OR "Athletic Performance"[Mesh] OR Motivation[Mesh]) |
| SPORTDiscus | ((TI "Rugby*" OR AB "Rugby*") OR (TI "Soccer" OR AB "Soccer") OR (TI "Football" OR AB "Football") OR (TI "oztag" OR AB "oztag") OR (TI "futsal" OR AB "futsal") OR (TI "lacrosse" OR AB "lacrosse") OR (TI "hockey" OR AB "hockey") OR (TI "hurling" OR AB "hurling") OR (TI "Water Polo" OR AB "Water Polo") OR (TI "Basketball" OR AB "Basketball") OR (TI "Netball" OR AB "Netball") OR (TI "Handball" OR AB "Handball") OR DE "Rugby" OR DE "Soccer" OR DE "Football" OR DE "Hockey" OR DE "Basketball")  AND  ((TI "coach*" OR AB "coach*") OR (TI "teach*" OR AB "teach*") OR (TI "mentor" OR AB "mentor") OR (TI "train*" OR AB "train*") OR (TI "learn*" OR AB "learn*") OR (TI "player" OR AB "player") OR DE "Teaching" OR DE "Mentors" OR DE "Learning" OR DE "Physical Education and Training" OR DE  athletes")  AND  ((TI "non-linear pedagog*" OR AB "non-linear pedagog*") OR (TI "non linear pedagog*" OR AB "non linear pedagog*") OR (TI "nonlinear pedagog*" OR AB "nonlinear pedagog*") OR (TI "constraints-led approach" OR AB "constraints-led approach") OR (TI "constraints led approach" OR AB "constraints led approach") OR (TI "indirect instruct*" OR AB "indirect instruct*") OR (TI "nonlinear dynamic" OR AB "nonlinear dynamic") OR (TI "non-linear dynamic" OR AB "non-linear dynamic") OR (TI "linear pedagog*" OR AB "linear pedagog*") OR (TI "direct instruct*" OR AB "direct instruct*") OR (TI "linear" OR AB "linear") OR (TI "nonlinear" OR AB "nonlinear") OR (TI "traditional" OR AB "traditional"))  AND  ((TI "skill*" OR AB "skill*") OR (TI "competence" OR AB "competence") OR (TI "capability" OR AB "capability") OR (TI "ability" OR AB "ability") OR (TI "perform*" OR AB "perform*") OR (TI "develop*" OR AB "develop*") OR (TI "precision" OR AB "precision") OR (TI "proficiency" OR AB "proficiency") OR (TI "technical" OR AB "technical") OR (TI "tactical" OR AB "tactical") OR (TI "mechanism" OR AB "mechanism") OR (TI "motivation" OR AB "motivation") OR (TI "prepar*" OR AB "prepar*") OR (TI "readiness" OR AB "readiness") OR DE "Motor Skills" OR DE "Athletic Performance" OR DE "Motivation") |
| OVID Medline | (Rugby*.tw. OR Soccer.tw. OR Football.tw. OR oztag.tw. OR futsal.tw. OR lacrosse.tw. OR hockey.tw. OR hurling.tw. OR "Water Polo".tw. OR Basketball.tw. OR Netball.tw. OR Handball.tw. OR exp Rugby/ OR exp Soccer/ OR exp Football/ OR exp Hockey/ OR exp Basketball/)  AND  (coach*.tw. OR teach*.tw. OR mentor.tw. OR train*.tw. OR learn*.tw. OR player.tw. OR exp Teaching/ OR exp Mentors/ OR exp Learning/ OR exp "Physical Education and Training"/ OR exp athletes/)  AND  ("non-linear pedagog*".tw. OR "non linear pedagog*".tw. OR "nonlinear pedagog*".tw. OR "constraints-led approach".tw. OR "constraints led approach".tw. OR "indirect instruct*".tw. OR "nonlinear dynamic".tw. OR "non-linear dynamic".tw. OR "linear pedagog*".tw. OR "direct instruct*".tw. OR linear.tw. OR nonlinear.tw. OR traditional.tw.)  AND  (skill*.tw. OR competence.tw. OR capability.tw. OR ability.tw. OR perform*.tw. OR develop*.tw. OR precision.tw. OR proficiency.tw. OR technical.tw. OR tactical.tw. OR mechanism.tw. OR motivation.tw. OR prepar*.tw. OR readiness.tw. OR exp "Motor Skills"/ OR exp "Athletic Performance"/ OR exp Motivation/) |
| CINAHL | ((TI Rugby* OR AB Rugby*) OR (TI Soccer OR AB Soccer) OR (TI Football OR AB Football) OR (TI oztag OR AB oztag) OR (TI futsal OR AB futsal) OR (TI lacrosse OR AB lacrosse) OR (TI hockey OR AB hockey) OR (TI hurling OR AB hurling) OR (TI "Water Polo" OR AB "Water Polo") OR (TI Basketball OR AB Basketball) OR (TI Netball OR AB Netball) OR (TI Handball OR AB Handball) OR (MH Rugby+) OR (MH Soccer+) OR (MH Football+) OR (MH Hockey+) OR (MH Basketball+))  AND  ((TI coach* OR AB coach*) OR (TI teach* OR AB teach*) OR (TI mentor OR AB mentor) OR (TI train* OR AB train*) OR (TI learn* OR AB learn*) OR (TI player OR AB player) OR (MH Teaching+) OR (MH Mentors+) OR (MH Learning+) OR (MH "Physical Education and Training+") OR (MH athletes+))  AND  ((TI "non-linear pedagog*" OR AB "non-linear pedagog*") OR (TI "non linear pedagog*" OR AB "non linear pedagog*") OR (TI "nonlinear pedagog*" OR AB "nonlinear pedagog*") OR (TI "constraints-led approach" OR AB "constraints-led approach") OR (TI "constraints led approach" OR AB "constraints led approach") OR (TI "indirect instruct*" OR AB "indirect instruct*") OR (TI "nonlinear dynamic" OR AB "nonlinear dynamic") OR (TI "non-linear dynamic" OR AB "non-linear dynamic") OR (TI "linear pedagog*" OR AB "linear pedagog*") OR (TI "direct instruct*" OR AB "direct instruct*") OR (TI linear OR AB linear) OR (TI nonlinear OR AB nonlinear) OR (TI traditional OR AB traditional))  AND  ((TI skill* OR AB skill*) OR (TI competence OR AB competence) OR (TI capability OR AB capability) OR (TI ability OR AB ability) OR (TI perform* OR AB perform*) OR (TI develop* OR AB develop*) OR (TI precision OR AB precision) OR (TI proficiency OR AB proficiency) OR (TI technical OR AB technical) OR (TI tactical OR AB tactical) OR (TI mechanism OR AB mechanism) OR (TI motivation OR AB motivation) OR (TI prepar* OR AB prepar*) OR (TI readiness OR AB readiness) OR (MH "Motor Skills+") OR (MH "Athletic Performance+") OR (MH Motivation+)) |
| OVID PsycInfo | (Rugby*.ti,ab. OR Soccer.ti,ab. OR Football.ti,ab. OR oztag.ti,ab. OR futsal.ti,ab. OR lacrosse.ti,ab. OR hockey.ti,ab. OR hurling.ti,ab. OR "Water Polo".ti,ab. OR Basketball.ti,ab. OR Netball.ti,ab. OR Handball.ti,ab. OR exp Rugby/ OR exp Soccer/ OR exp Football/ OR exp Hockey/ OR exp Basketball/)  AND  (coach*.ti,ab. OR teach*.ti,ab. OR mentor.ti,ab. OR train*.ti,ab. OR learn*.ti,ab. OR player.ti,ab. OR exp Teaching/ OR exp Mentors/ OR exp Learning/ OR exp "Physical Education and Training"/ OR exp athletes/)  AND  ("non-linear pedagog*".ti,ab. OR "non linear pedagog*".ti,ab. OR "nonlinear pedagog*".ti,ab. OR "constraints-led approach".ti,ab. OR "constraints led approach".ti,ab. OR "indirect instruct*".ti,ab. OR "nonlinear dynamic".ti,ab. OR "non-linear dynamic".ti,ab. OR "linear pedagog*".ti,ab. OR "direct instruct*".ti,ab. OR linear.ti,ab. OR nonlinear.ti,ab. OR traditional.ti,ab.)  AND  (skill*.ti,ab. OR competence.ti,ab. OR capability.ti,ab. OR ability.ti,ab. OR perform*.ti,ab. OR develop*.ti,ab. OR precision.ti,ab. OR proficiency.ti,ab. OR technical.ti,ab. OR tactical.ti,ab. OR mechanism.ti,ab. OR motivation.ti,ab. OR prepar*.ti,ab. OR readiness.ti,ab. OR exp "Motor Skills"/ OR exp "Athletic Performance"/ OR exp Motivation/) |
