# Supplementary material for: The Effectiveness of Linear and Nonlinear Pedagogical Approaches in Team-Invasion Ball Sports: A Systematic Review
Source: Sports Med Open. 2025 Aug 4;11:90. doi: 10.1186/s40798-025-00893-y (PMC12321719; doi:10.1186/s40798-025-00893-y)
Supplement: Supplementary file 2 — Additional file 2. [file 40798_2025_893_MOESM2_ESM.docx]

**Supplementary Information File 2- Excluded Studies**

The effectiveness of linear and nonlinear pedagogical approaches in team-invasion ball sports: A systematic review

Journal of Sports Medicine

Liam Bromilow^1^, Nikki Milne^1^, Carl T. Woods^2,3^, Caroline Dowsett^1^ and Justin W. L Keogh^1,4,5^

^1^ Faculty of Health Sciences and Medicine, Bond University, Gold Coast, Australia

^2^ Institute for Health and Sport, Victoria University, Melbourne, Australia

^3^ Sport and Exercise Science, College of Healthcare Sciences, James Cook University

^4^ Sports Performance Research Centre New Zealand, Auckland University of Technology, Auckland, New Zealand

^5^ Kasturba Medical College, Mangalore, Manipal Academy of Higher Education, Manipal, Karnataka, India

Correspondence

Liam Bromilow: [liam.bromilow@student.bond.edu.au](mailto:liam.bromilow@student.bond.edu.au); ORCID: 0009-0007-7643-8454

Nikki Milne: 0000-0002-5121-9825

Carl Woods: 0000-0002-7129-8938

Caroline Dowsett: 0000-0001-7734-9436

Justin Keogh: 0000-0001-9851-1068

Supplementary Information File 2 – Justification of Excluded Studies

| **Wrong Intervention** | **Non-English Language** | **Wrong Outcomes** | **Wrong Comparator** | **Wrong Study Design** | **Non-Peer Reviewed Study** | **Full-Text Not Available** |
| --- | --- | --- | --- | --- | --- | --- |
| Al-Badr et al., 2020  Alhayek et al., 2003  Asadi et al., 2023  Ashraf et al., 2017  Ashraf et al., 2017  Browne et al., 2004  Cieslicka et al., 2019  Dania et al., 2020  Dicks et al., 2017  Garcia-Ceberino et al., 2019  Gonzalez-Espinosa et al., 2020  Gonzalez-Espinosa et al., 2021  Gray et al., 2011  Greco et al., 2010  Gurbuz et al., 2023  Hodges et al., 2006  Hohmann et al., 2016  Hooper et al., 2008  Huang et al., 2023  Jiang et al., 2022  Jing et al., 2023  Lopez et al., 2016  Miller et al., 2017  Nathan et al., 2017  Oboeuf et al., 2020  Oftadeh et al., 2021  Osman et al., 2016  Razak et al., 2020  Rocamora et al., 2019  Sierra-Rios et al., 2020  Tallir et al., 2005  Tallir et al., 2007  Tallir et al., 2012  Valencia Sanchez et al., 2021  van Slooten et al., 1976  Zhang et al., 2023  Zago et al., 2016 | Abdelaleem et al., 2023  Abu-Halima et al., 1995  Alizadeh et al., 2021  Eroglu et al., 2018  Flores-Rodriguez et al., 2021  Gharayagh Zandi et al., 2020  Hernandez et al., 2023  Ibanez Godoy et al., 2016  Kreivyte et al., 2009  Lubshf et al., 2020  Mardiny et al., 2003  Pascual Verdu et al., 2017 | Abdullah et al., 2020  Amani-Shalamzari et al., 2019  Burgueno et al., 2017  Gonzalez-Artetxe et al., 2022  Gonzalez-Espinosa et al., 2020  Sporis et al., 2008 | Eather et al., 2021  Memmert et al., 2007  Pizarro et al., 2019 | Kraemer et al., 2015  Light et al. 2014  Russell et al., 1993 | Angarola et al., 2005  Arias et al., 2017 | Ma et al., 2019 |
